# Bioclimatic distribution and prevalence maps for *Fasciola hepatica* in Espírito Santo State, Brazil

**DOI:** 10.1186/1678-9199-20-32

**Published:** 2014-07-29

**Authors:** Deivid França Freitas, Isabella VF Martins, Gleissy MADA dos Santos, Alexandre R dos Santos, Daniel da Silva Gomes

**Affiliations:** 1 Graduate Program in Veterinary Sciences, School of Veterinary Sciences, Federal University of Espírito Santo, Alegre, Espírito Santo State, Brazil; 2 Graduate Program in Pharmaceutical Sciences, School of Pharmaceutical Sciences, University of Vila Velha, Vila Velha, Espírito Santo State, Brazil; 3 Graduate Program in Forest Sciences, School of Forest Sciences, Federal University of Espírito Santo, Alegre, Espírito Santo State, Brazil; 4 Fluminense University Center (UNIFLU), Campos dos Goytacazes, Rio de Janeiro State, Brazil; 5 Centro de Ciências Agrárias, Universidade Federal do Espírito Santo, Alegre, Espírito Santo, Brasil

**Keywords:** Maps, Geographic information system (GIS), Prevalence, *Fasciola hepatica*

## Abstract

**Background:**

Fasciolosis affects different ruminant species and leads to great economic losses for cattle farmers worldwide. Thus, the current study aimed to evaluate bovine fasciolosis prevalence in the state of Espírito Santo, Brazil, using slaughter maps provided by slaughterhouses and verifying the origin of cattle.

**Methods:**

A map was created based on analysis of epidemiological data. The ArcGIS/ArcINFO 10.1 software was employed in order to elaborate updated bioclimatic maps that displayed the fasciolosis prevalence within the state – per city– between 2009 and 2011.

**Results:**

According to the bioclimatic map it was clear that 52.24% of the state’s total area comprise regions considered favorable for the development and survival of *Fasciola hepatica*. According to the data provided by slaughterhouses, the parasite was more frequent in the cities of Atílio Vivácqua, Itapemirim and Anchieta with respective prevalence of 28.41, 25.50 and 24.95%. Although the northern portion of the state is also favorable for the disease maintenance (reaching rates above 90%), several cities presented prevalence of only 0.99 and 1.94% respectively. These findings indicate that climatic and environmental factors only cannot be considered preponderant to fasciolosis occurrence. Regarding the slaughterhouse located in Anchieta city, the higher prevalence was registered in the cities of Jerônimo Monteiro, Alegre and Cachoeiro de Itapemirim, with mean prevalence of 1.21, 1.07 and 2.09% respectively.

**Conclusion:**

Although the present findings suggest a pattern for the prevalence of fasciolosis, records of the cities for the occurrence of the disease usually do not reflect the true origin of animals.

## Background

Fasciolosis is a parasitosis that affects cows, buffalos, donkeys, sheep and occasionally human beings [1]. Originally from Europe, the trematoda *Fasciola hepatica* successfully increased its geographic boundaries and nowadays can be found in all the continents. Besides restrictions imposed by its biology and ecology, the parasite holds a cosmopolitan distribution that ranges from temperate regions up to the tropics [2].

In following up the impact of fasciolosis in cattle farming around the world, it is noticeable that economic losses are considerable once they can overpass US$ 2 billion a year. Such losses comprise decrease in dairy production, reduction in animal weight, slow development and fertility impairment, besides the discard of a large number of infected livers found in slaughterhouses [3].

Due to its strong dependence on weather and environment, it is observed that the distribution and prevalence of the disease is higher in places where mean temperatures vary from 10°C to a little more than 25°C [4].

Factors such as rainfall, air relative humidity, soil conditions and flooded areas are important for the development and survival of the parasite *F. hepatica.* It is also known that flooded lands are necessary to the occurrence of aquatic mollusks from the genus *Lymnaea –* intermediate hosts of the larval stages of trematoda [5].

The first reports, in Brazil, about the disease came from the state of Rio de Janeiro around 1920 [6]. Since then, cases of fasciolosis have often been recorded throughout the country. The disease have been registered mainly in the South and Southeast, due to high infection indexes observed in these areas [7,8].

Based on these records, the existence of possible risk areas for fasciolosis in the South of the state of Espírito Santo was hypothesized [9]. Martins *et al.*[10] found eggs of the parasite in feces of animals slaughtered in this region when they were evaluating the efficiency of a sedimentation technique.

As new technologies arose, the use of geographic information systems (GIS) became mandatory because of their contributions to cartographic data, particularly in spatial and temporal epidemiological models used to categorize transmission risks of certain diseases [11].

Bioclimatic risk is highlighted among the mechanisms developed by GIS. It aims to quantify risks according to the background of adverse events based on epidemiology and local climate. It is known that distribution is a broadly applied technique to identify regions most favorable to the development of many cultures [12]. Therefore, it is worth using such technique as a veterinarian epidemiology tool, once the dispersion and establishment of many parasitic diseases are deeply linked to environmental and climatic factors.

Thus, the present study aimed to elaborate a bioclimatic distribution for *F. hepatica* in the state of Espírito Santo using GIS and data about the prevalence of bovine fasciolosis provided by slaughterhouses registered at the federal and state inspection systems (SIF and SIE) of the Brazilian Ministry of Agriculture, Livestock and Supply (MAPA).

## Methods

### Studied location

The state of Espírito Santo holds a 46.184,1 km^2^ area divided into 78 cities and it is geographically located between meridians 39°38’ and 41°50’ west longitude and between parallels 17°52’ and 21°19’ south latitude.

Mean altitude is 600–700 m and presents quite rough topography, archeozoic lands with isolated peaks about 2.890 m high. The edge of the highlands presents itself as a mountain zone and it is crossed by rivers that open deep valleys, which creates flooding zones during rainfall periods [13].

It is known that Espírito Santo holds two main climatic types: tropical rainy and humid mesothermal. The first one dominates the lowlands and is characterized by high temperatures throughout the year and average temperature above 22°C. The humid mesothermal climate – which has no dry season – is found in mountain areas in the south of the state. It is characterized by low temperatures in the winter and medium in the colder months (bellow 18°C). According to Köppens’ classification, the climate is *Aw*-warm and humid (tropical rainy), presenting rain in summer and drought in winter (sub-drought in August) [14].

### Bioclimatic research for the occurrence of fasciolosis

Data recorded during 30 years were used to elaborate distribution maps for fasciolosis in the state of Espírito Santo. The information was dereived from 109 weather stations located in the state and bordering areas, which favored data interpolations and the application of the hydric balance according to Thornthwaite and Matter [15].

### Statistical analysis and cartographic data generation

After data analysis using the ArcGIS/ArcINFO 10.1 software, charts of hydric balance were imported to generate punctual spatial vectors from meteorological stations and their respective attribute tables. Afterwards, the multiple linear regression was applied. It utilized altitude and geographic coordinates – longitude (X) and latitude (Y) – as independent variables of Mercator Transverse Universal System (UTM). Temperature was the dependent variable. Data were made available in attribute tables of hydric balance, as described below, by the multiple regression equation:

$$T=\beta_{0}+\beta_{1}\mathrm{ALT}+\beta_{2}X+\beta_{3}Y$$

In which:

T: temperature (°C);

ALT: altitude (m);

X: coordinates UTM X (m);

Y: coordinates UTM Y (m);

β_0_: constant of regression;

β_1_, β_2_ and β_3_: coefficient of regression for the variables ALT, X and Y.

Thus, matrix images of longitude (X) and latitude were generated by means of a spatial interpolation using the Spherical Kriging Method and imported into the application ArcGIS/ArcINFO 10.1. The Elevation Digital Model (EDM) from the Shuttle Radar Topography Mission (SRTM) project was also generated and made available by the Brazilian Enterprise for Agricultural Research (EMBRAPA) following the scale 1:250.000 in the cartographic projection WGS84.

Four variables featuring the distribution of fasciolosis were developed by means of a mathematical model combined with GIS. They were inserted in a database according to their relevance to the disease. Therefore, weights varying from 1 to 3, were adopted and divided into areas that presented high, intermediate and low favorability for fasciolosis in Espírito Santo.

The spatial reclassification was applied to matrix images of altitude, temperature and rainfall in order to highlight regions of capability, restrictiveness and infeasibility, as shown in Table 1.

**Table 1:** Attributes and their relative weights according to the potential risk of fasciolosis in Espírito Santo state, Brazil

**Variable**	**Relative weight**		
	**1 – Suitable**^**1**^	**2 – Restrictive**^**1**^	**3 – Impracticable**^**1**^
Altitude	0-500 m	500-1000	>1000 m
Temperature	15-25°C	10-15°C and > 25°C	< 10°C
Slope	Until 10%	10 to 15%	Above 15%
Rainfall	1000-2500 mm/year	> 1000 mm/year	> 2500 mm/year

^1^(1) Suitable region, (2) restrictive region and (3) impracticable region for the occurrence of fasciolosis.

Such values were based on studies that have assumed infeasible temperatures: below 10°C, restricted to 10-15°C and higher than 25°C. They also considered optimum temperatures – from 10°C up to 26°C, besides favorable altitudes, those set between 0 and 500 m above sea level, as well as steepness values up to 10% [16,17]. It is known that favorable altitudes might be higher than 1000 m, as in cases of fasciolosis reported in the Andes [18]. The current study made an option for rates that better represent local conditions.

The “cross tabulating” function was applied in order to develop a bioclimatic distribution able to meet the occurrence of fasciolosis. Thus, matrix images representing the bioclimatic division were converted into a polygonal vector format. The “polygonal dissolution” function was applied in order to obtain the vector image, due to the large numbers of polygons.

The “geometric calculation” function was applied to calculate the respective areas (km^2^) and perimeters (km^2^) of the mentioned aptitude classes. It was done by using attributes resulting from polygonal vector images found in the GIS environ.

### Bioclimatic distribution for the 78 cities

In order to develop an individual bioclimatic map for each one of the 78 counties in the state, the “intersection” function was applied to polygonal vector images from the cities and to distribution that totalized two new polygonal vector images representing the bioclimatic zone in each city.

### Collecting data from *Fasciola hepatica* infection

Data about discarded bovine livers from animals slaughtered throughout the state of Espírito Santo were made available by means of slaughter maps showing infection rates of *F. hepatica* in 11 slaughterhouses registered at the federal and state inspection systems (SIF and SIE) of the Brazilian Ministry of Agriculture, Livestock and Supply between 2009 and 2011. The aforementioned scenario took the location of slaughterhouses into acccount. At the same time, only a slaughterhouse in Anchieta city – south of Espírito Santo – provided data about the origin of animals. The existing correlation found between aptitude areas and the respective mean fasciolosis prevalence in Espírito Santo was calculated by means of the Pearson correlation coefficient with significance level of 95%.

### Developing the final prevalence maps

**Figure 1:**
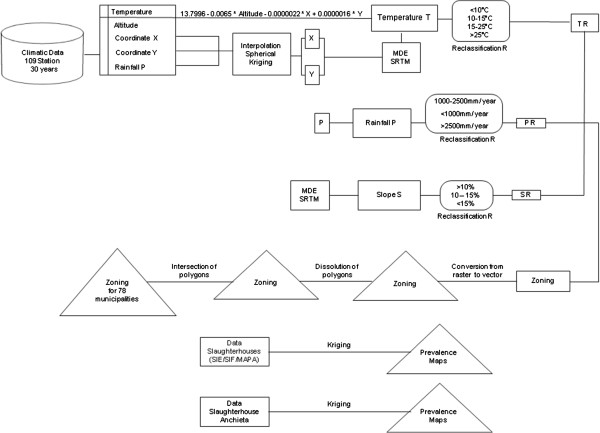
Flowchart statement about the steps performed for the generation of the bioclimatic distribution and prevalence maps.

Data provided by slaughterhouses (SIF and SIE) as well as information about the origin of animals from the slaughterhouse in Anchieta city were used in order to develop bovine fasciolosis prevalence maps in the state of Espírito Santo. The ordinary spherical Kriging technique was used to generate cartographic maps. According to Landim [19], the method tries to minimize estimated variances based on preview regression models. He took into account the stochastic dependency among data geospatially distributed.All the procedures required by the methodology are represented in Figure 1.

## Results

### Prevalence of fasciolosis

**Figure 2:**
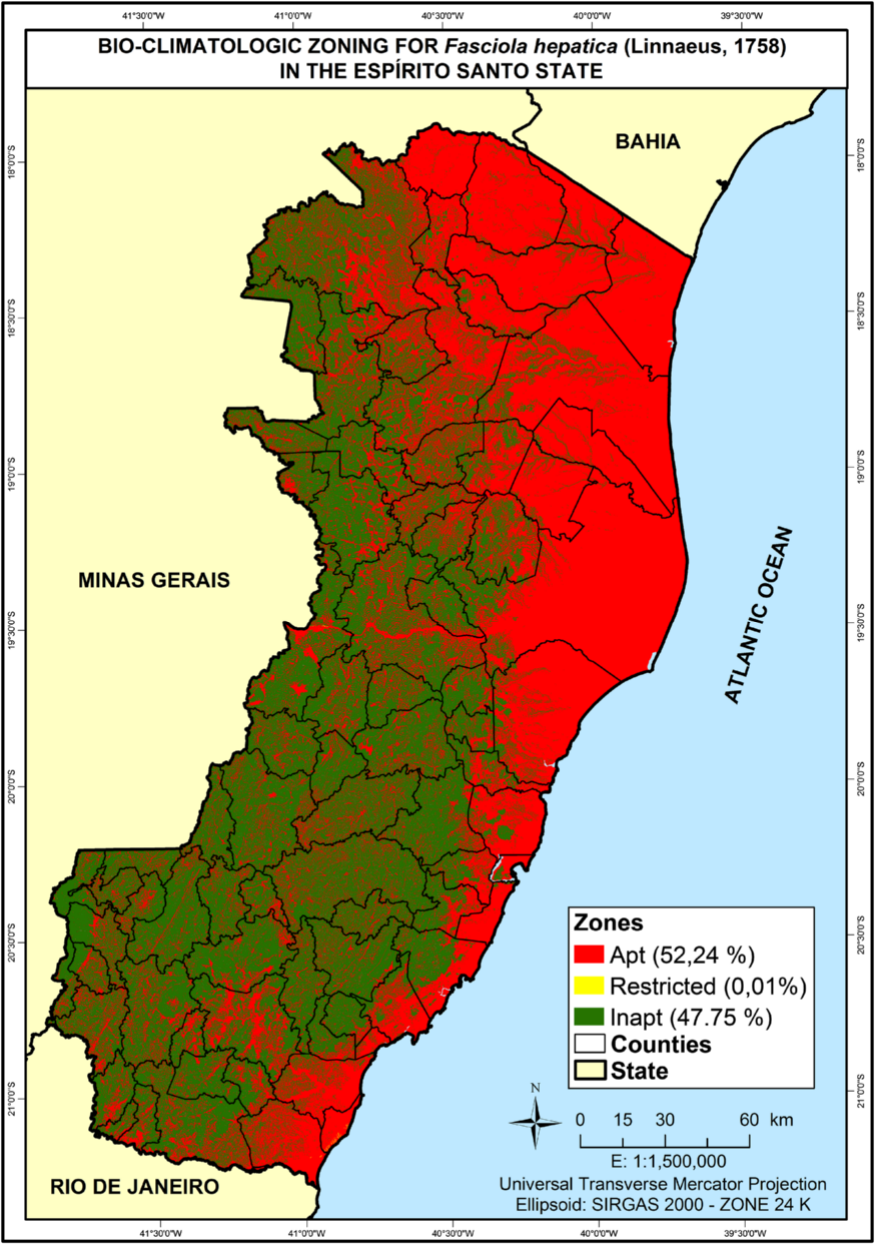
**Bioclimatic distribution of *F*. *hepatica *** **in Espírito Santo and suitability for its development in the state.**

The bioclimatic map for the state of Espítito Santo – which was based on temperature, rainfall, altitude and steepness variables (Figure 2) – had shown that 52.25% of areas within the state are located in regions that favor the development of *Fasciola hepatica* and its intermediate hosts whereas 47.75% of the areas are not suitable for them.

**Figure 3:**
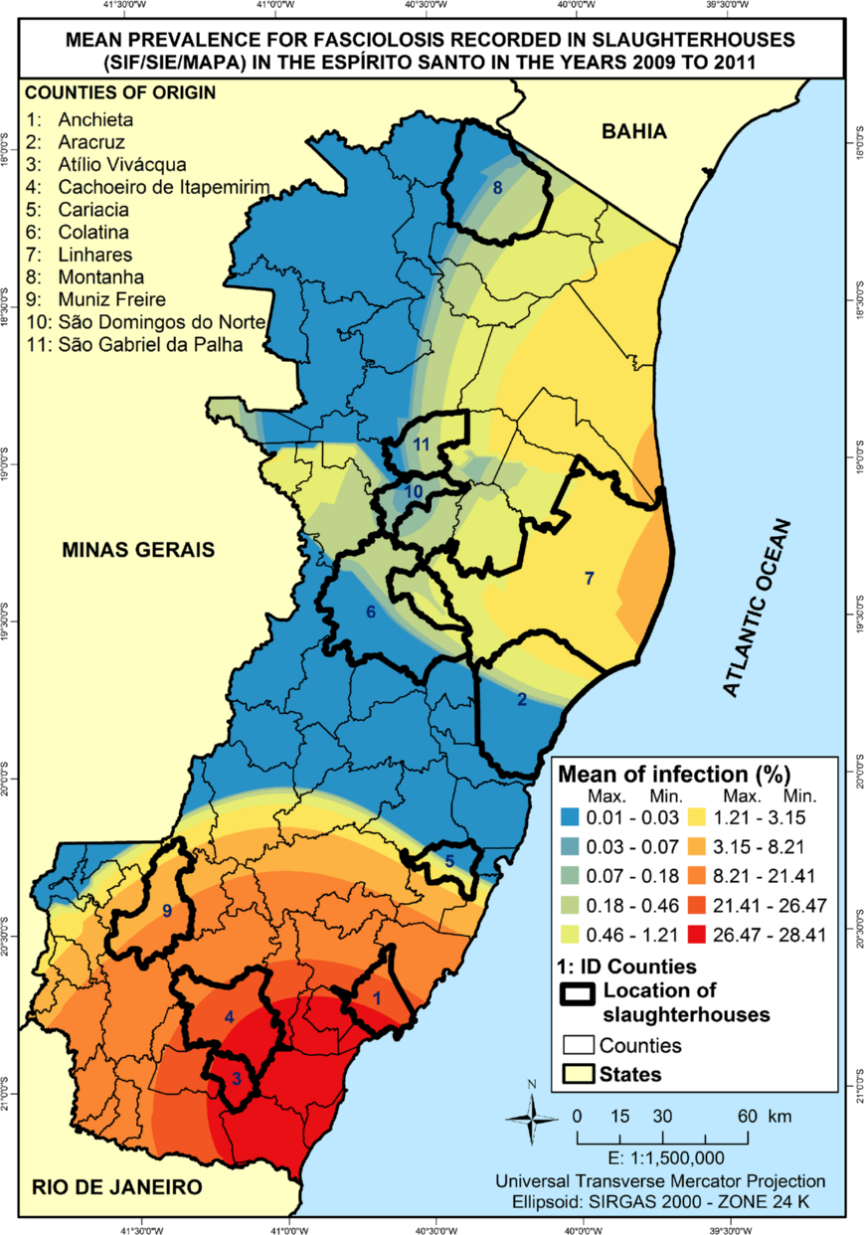
Map showing the prevalence of bovine fasciolosis in the state of Espírito Santo from 2009 to 2011.

Therefore, maps generated by distribution, based on rates of prevalence from 11 slaughterhouses distributed throughout the state and monitored by SIF and SIE were combined. Results indicated that slaughterhouses in Atílio Vivácqua, Cachoeiro de Itapemirim and Anchieta presented higher rates of discarded livers, with mean prevalence of 28.41, 25.50 and 24.95% respectively (Table 2).Regarding data presented in Figure 3, it was observed that maps generated by distribution showed areas with higher significance for the occurrence of the disease in the south of the state. They presented average rates that varied from 1.21 to 28.41%.

**Table 2:** Environmental suitability for bovine fasciolosis and its mean prevalence from 2009 to 2011 regarding distribution by counties in Espírito Santo state according to slaughter maps provided by the Institute of Agricultural and Forestry Defense of Espírito Santo State (IDAF)

**Cities**	**Area (km**^**2**^**)**	**Suitability (%)**		**Mean prevalence (%)**			
		**Suitable**	**Unsuitable**	**2009**	**2010**	**2011**	**3 years**
Anchieta	405.32	65.06	34.94	27.64	25.94	21.27	24.95
Aracruz	1 435.81	89.07	10.93	0.04	0.00	0.00	0.01
Atílio Vivácqua	226.86	33.42	66.58	28.25	28.04	28.93	28.41
Cachoeiro de Itapemirim	877.31	39.56	60.44	–	–	25.50*	25.50
Cariacica	275.74	52.55	47.45	0.68	0.00	0.00	0.23
Colatina	1 426.02	33.98	66.02	0.03	0.02	0.04	0.03
Linhares	3 500.19	90.71	9.29	0.00	0.00	5.83	1.94
Montanha	1 096.65	94.81	5.19	1.80	0.89	0.30	0.99
Muniz Freire	679.78	16.41	83.59	4.63	7.07	10.55	7.42
São Domingos do Norte	299.80	45.27	54.73	0.04	0.00	0.05	0.03
São Gabriel da Palha	434.83	47.23	52.77	0.26	0.42	0.17	0.28

*The slaughterhouse in Cachoeiro de Itapemirim city only began operations in 2011.

In the extreme North of the state, Montanha showed 0.99% of prevalence. Although this city is within the range aptitude for the disease, that is 94.81%, it did not register any higher prevalence than the average found during the three years of the study (Table 2).

### Prevalence of fasciolosis in a slaughterhouse in Anchieta city, southern Espírito Santo

**Figure 4:**
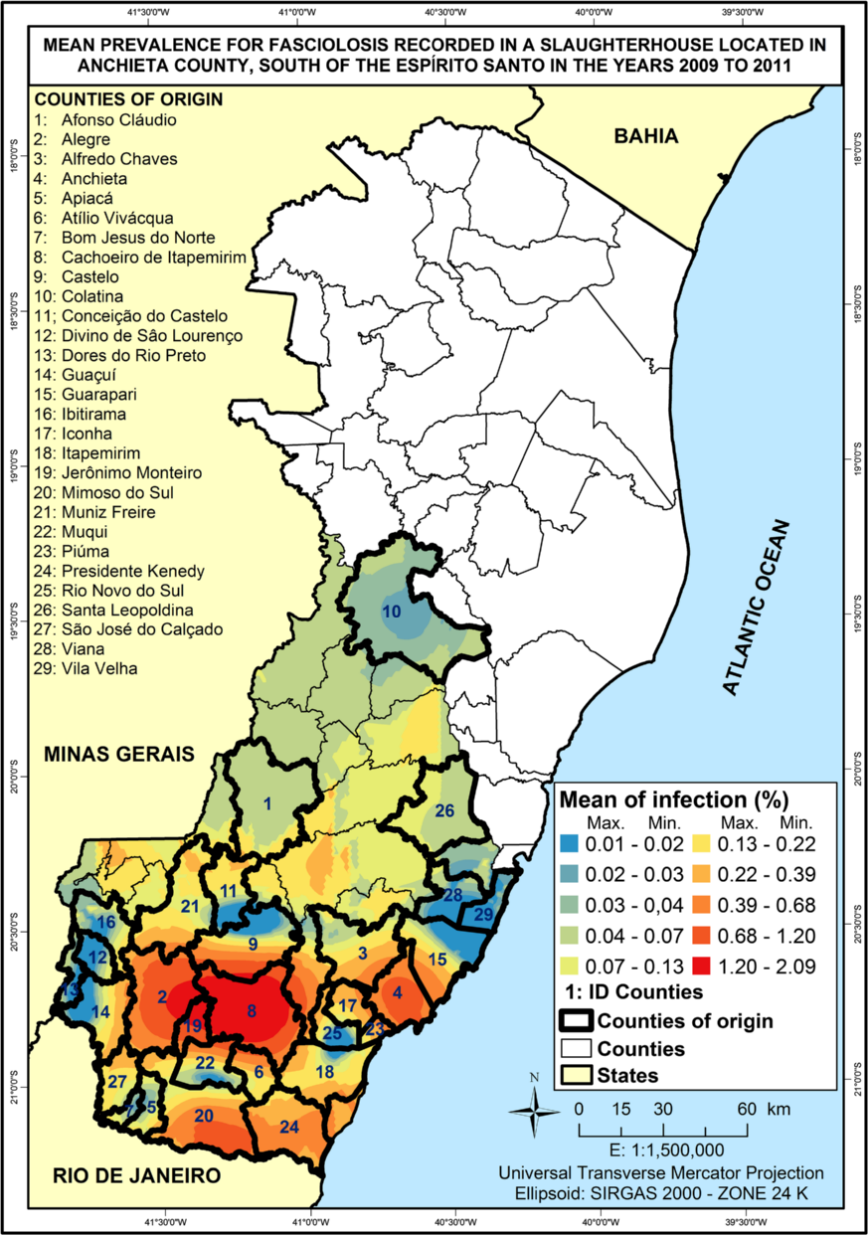
Map showing the prevalence of bovine fasciolosis and its origins in Espírito Santo from 2009 to 2011 according to data provided by a slaughterhouse located in Anchieta.

The geospatial distribution of mean prevalence rates recorded in the slaughterhouse of Anchieta, as aforementioned, indicated high prevalence for fasciolosis in municipalities situated in the southern region of the state, with values ranging from 0.01 to 2.09% (Figure 4). The cities that stood out were Jerônimo Monteiro, Alegre and Cachoeiro de Itapemirim, with averages of 1.21, 1.07 and 2.09% respectively (Table 3).

**Table 3:** Suitability for bovine fasciolosis and mean prevalence between 2009 and 2011 according to its origin by cities in the Espírito Santo state

**Counties**	**Area (km**^**2**^**)**	**Suitability (%)**	**Mean prevalence (%)**					
	**Area**	**Suitable**	**Unsuitable**	**Restrict**	**2009**	**2010**	**2011**	**3 years**
Afonso Cláudio	955.26	19.24	80.76	0.00	0.14	0.00	0.01	0.05
Alegre	773.40	23.71	76.29	0.00	1.06	1.47	0.68	1.07
Alfredo Chaves	616.57	17.99	82.01	0.00	0.00	0.32	0.12	0.15
Anchieta	405.32	65.06	34.94	0.00	0.29	1.35	0.74	0.79
Apiacá	193.67	37.39	62.61	0.00	0.03	0.09	0.00	0.04
Atílio Vivácqua	226.86	33.42	66.58	0.00	0.03	0.49	0.16	0.23
Bom Jesus do Norte	89.67	33.10	66.90	0.00	0.14	0.00	0.00	0.05
Cachoeiro de Itapemirim	877.31	39.56	60.44	0.00	2.34	1.44	2.50	2.09
Castelo	668.98	21.63	78.37	0.00	0.00	0.03	0.49	0.17
Colatina	1 426.02	33.98	66.02	0.00	0.00	0.00	0.05	0.02
Conceição do Castelo	364.82	20.82	79.18	0.00	0.19	0.12	0.08	0.13
Divino de São Lourenço	175.81	26.92	73.08	0.00	0.00	0.04	0.00	0.01
Dores do Rio Preto	152.78	21.03	78.97	0.00	0.09	0.01	0.00	0.03
Guaçuí	468.88	31.72	68.28	0.00	0.03	0.22	0.00	0.08
Guarapari	595.07	50.00	50.00	0.00	0.03	0.20	0.12	0.11
Ibitirama	330.00	21.42	78.58	0.00	0.00	0.03	0.00	0.01
Iconha	202.61	19.11	80.89	0.00	0.06	0.51	0.20	0.26
Itapemirim	557.97	76.99	23.01	0.00	0.14	0.12	0.12	0.13
Jerônimo Monteiro	162.31	35.36	64.64	0.00	1.36	1.58	0.65	1.21
Mimoso do Sul	869.08	32.73	67.27	0.00	0.43	0.52	0.51	0.50
Muniz Freire	679,78	16.41	83.59	0.00	0.03	0.14	0.18	0.12
Muqui	327.47	19.18	80.82	0.00	0.00	0.32	0.00	0.11
Piúma	74.08	84.98	15.02	0.00	0.24	0.46	0.27	0.32
Presidente Kenedy	586.99	78.37	21.59	0.04	0.25	1.02	0.04	0.43
Rio Novo do Sul	203.78	30.15	69.85	0.00	0.01	0.10	0.05	0.05
Santa Leopoldina	272.78	21.93	78.07	0.00	0.06	0.08	0.08	0.07
São José doCalçado	715.68	27.39	72.61	0.00	0.17	0.17	0.16	0.17
Viana	312.19	41.10	58.90	0.00	0.01	0.04	0.01	0.02
Vila Velha	210.71	95.87	4.13	0.00	0.00	0.03	0.00	0.01

Data provided by a slaughterhouse in Anchieta city, in the south of the state.

Regarding the distribution of cities based on the origin of positive animals, the correlation between capable areas and prevalence rates was 0.09. It was considered a weak correlation.

## Discussion

For the elaboration of a plan of effective control against fasciolosis it is necessary an epidemiological approach that takes into account local and regional issues, in order to reduce the use of anthelmintics, and that corroborates the idea of an integrated management control based on the rational use of products available in the market, which also monitors the emergence of resistance. Thus, the use of geographic information systems agrees with the current needs of the biotech market and veterinary medicine, since it is seen as a preventive tool that helps the producer to make decisions aiming for reduction of losses.

It is noticed the existence of a pattern in prevalence data from slaughterhouses. The origin of animals is not always taken into account and is limited to the reports of slaughterhouses. The present work aimed to demonstrate data on the prevalence of fasciolosis – provided by slaughterhouses registered at federal and state inspection systems and by a slaughterhouse in the southern region of the state – in order to offer a better understanding on the source of the infected animals.

The maps displayed in the present study were generated based on bioclimatic distribution, in which suitable areas are located in zones considered favorable to the development of *Fasciola hepatica* and its intermediate hosts, given that mean temperature and annual rainfall in such places meet limits and are considered positive for the parasite survival and maintenance. They present temperature variations around 25°C or more and rainfall averages between 1000 and 2500 mm/year [20].

It is worth highlighting – based on information from maps generated by distribution and mean prevalence rates from slaughterhouses located in Atílio Vivácqua, Cachoeiro de Itapemirim and Anchieta – that despite the fact that Cachoeiro de Itapemirim city presented elevated percentages (Table 2), the local slaughterhouse initiated its activities in 2011. Such findings corroborate studies performed by Bernardo *et al.*[21]. They reported 24.89% prevalence of fasciolosis in a slaughterhouse in the south of Espírito Santo. Martins *et al.*[10], who tested the sensitivity of a previously reported technique in Atílio Vivácqua, found that 14.14% of the studied animals were parasitized by *F. hepatica.* Corroborating these results, Alves *et al.*[22] found the presence of *F. hepatica* in several rural properties located in the south of Espírito Santo state.

Similar results were also found by Dutra *et al.*[23] in the south of Brazil. They mentioned 29.51% of discarded livers in the following states: Santa Catarina, Paraná and Rio Grande do Sul. Other studies had already reported the presence of mollusks of the genus *Lymnaea* in considerably less extensive areas, but with higher prevalence rates. This species of mollusk is considered an important factor to the occurrence of the disease [9]. Bernard *et al*. [21] found high prevalence of fasciolosis in cities also situated in less suitable regions, which proves that even in these areas the biological factor, i. e. the presence of mollusks, may be fundamental for the occurrence of the disease.

After analyzing the north region of Espírito Santo, an information gap was observed regarding data collection procedures adopted by the Laboratory of Malacology of the Federal University of Espírito Santo for mollusks of the genus *Lymnaea*. Somehow, it indicates that such snails – intermediate hosts of *F. hepatica* – did not colonize or establish successful colonies in the region. Thus, the existence of positive cases of *F. hepatica* infection can be related to the transportation of animals to other cities and neighbor states such as Minas Gerais and Bahia [24].

The correlation between suitable areas in the state of Espírito Santo and fasciolosis prevalence was 0.31. Such result reinforces the hypothesis related to the transportation of animals from one region to another, trade among farmers and absence of intermediate hosts in more suitable places, keeping the prevalence low.

It is known that factors that influence the occurrence of fasciolosis in a certain region are connected to the availability of adequate biotopes to the development of snails. Therefore, the proportion of recorded infections in Montanha could be connected to the circulation of animals coming from other regions. Such regions usually present positive cases of the disease, even when there is no record of intermediate hosts in them [25,26].

The analysis of the central, northeast and northwest regions of the state showed that Linhares had an infection rate of 1.94%. It strongly influenced the distribution limits, once interpolation can lead to a tendency of growth in susceptible areas towards west. Prevalence rates from slaughterhouses in neighbor counties were considerably lower and had values varying from 0.01 up to 0.28%.

Such result is supported by Namikawa [27] who stated that local interpolator sites used for preparation of GIS maps lead to complete changes within local values, once spots closer to predominance limits are interpolated, thus generating an estimative of values of samples from a known number.

Martins *et al.*[9] described that the cities of Jerônimo Monteiro, Alegre and Cachoeiro de Itapemirim are favorable for the disease, since they are inserted into high risk areas with more than 50% of their territorial extensions suitable for *F. hepatica* development. These values of favorability are applicable to the present results, in which data provided by slaughterhouses show that percentages are higher in the southern region of the State.

In the current study, it was observed that the lowest rates of infection were found in the metropolitan areas, in Serra do Caparaó and mountainous regions with averages between 0.01 to 0.13% (Figure 4). These high altitude areas are composed of cities that have unfavorable conditions for the development of *F. hepatica* (Table 3).

Mas-Coma *et al*. [18] found the parasite *F. hepatica* and *Lymnaea* snails in the Bolivian highlands (more than 3800 m altitude). It is known that the transmission of the parasite is facilitated in low altitudes, because in high altitudes less oxygen is available, temperature is more extreme and solar radiation is elevated. Thus, animals raised in such places may show different morphological and physiological features from those living in low lands [28].

Mas-Coma *et al.*[18] observed that altitude effects are possibly softened by the solar incidence. These areas located in the tropics present absence of dense vegetation, porous soil and other factors. These variables could directly favor higher temperature and rainfall rates. Such factors combined with shallow water tables on the soil would consequently lead to permanent water backlog in such places.

Although Figure 3 showed that Colatina appeared among the places affected by fasciolosis, it is known that the city is located in a zone unsuitable for the parasite (Table 3). Colatina does not border areas with positive cases. It is interesting to observe that data from slaughterhouses showed that it can possibly be part of a region facing a fasciolosis outbreak (Figure 4). This case highlights that it is important to analyze data as a whole in order to a get a complete picture of the situation, which would positively influence the decision-making process regarding public healthcare policies.

## Conclusions

Considering the data provided by federal and state inspection systems (SIF and SIE), a slaughterhouse in Anchieta and generated maps, it can be observed that southern Espírito Santo showed the highest prevalence for fasciolosis, which corroborates previous studies.

Although collected data suggested a prevalence pattern based on bioclimatic maps, this observation was not true for several cities, particularly those in the north of the state. This may be attributable to a few facts including that data from slaughterhouses do not reflect the real origin of animals and to trade of animals among farmers.

Control strategies including stricter surveillance of animal transport by competent organs may effectively prevent the occurrence of new cases in regions considered free of the disease. Because when the presences of the parasite and its intermediate host are already observed, the control of fasciolosis becomes expensive and less effective.

## Competing interests

The authors declare that there are no competing interests.

## Authors’ contributions

IVFM: project coordinator. DFF: performed all the research. GMADAS, ARS and DSG: responsible for GIS and statistical analysis. All authors read and approved the final manuscript.
